# Redox Active Transition Metal ions Make Melanin Susceptible to Chemical Degradation Induced by Organic Peroxide

**DOI:** 10.1007/s12013-017-0793-6

**Published:** 2017-04-11

**Authors:** Andrzej Zadlo, Anna Pilat, Michal Sarna, Anna Pawlak, Tadeusz Sarna

**Affiliations:** Department of Biophysics, Faculty of Biochemistry, Biophysics and Biotechnology, Jagiellonian University, Krakow, Poland

**Keywords:** Melanin, Melanosomes, Transition metal ions, Organic peroxides, Oxidative degradation, Antioxidant

## Abstract

With aging, retinal pigment epithelium melanosomes, by fusion with the age pigment lipofuscin, form complex granules called melanolipofuscin. Lipofuscin granules may contain oxidized proteins and lipid hydroperoxides, which in melanolipofuscin could chemically modify melanin polymer, while transition metal ions present in melanin can accelerate such oxidative modifications. The aim of this research was to examine the effect of selected transition metal ions on melanin susceptibility to chemical modification induced by the water-soluble tert-butyl hydroperoxide used as an oxidizing agent. Synthetic melanin obtained by DOPA autooxidation and melanosomes isolated from bovine retinal pigment epithelium were analyzed. To monitor tert-butyl hydroperoxide-induced oxidative changes of DMa and BMs, electron paramagnetic resonance spectroscopy, UV-vis absorption spectroscopy, dynamic light scattering, atomic force microscopy and electron paramagnetic resonance oximetry were employed. These measurements revealed that both copper and iron ions accelerated chemical degradation induced by tert-butyl hydroperoxide, while zinc ions had no effect. Strong prooxidant action was detected only in the case of melanosomes and melanin degraded in the presence of iron. It can be postulated that similar chemical processes, if they occur in situ in melanolipofuscin granules of the human retinal pigment epithelium, would modify antioxidant properties of melanin and its reactivity.

## Introduction

Melanin is a photoprotective pigment present in plants and animals. Skin melanin efficiently screens solar radiation, especially its UV component [1]. In vitro research showed that melanin can also act as an antioxidant [2, 3]. In the retinal pigment epithelium (RPE), melanin shows very little, if any, metabolic turnover [4], suggesting that with ageing the RPE melanin may undergo chemical modifications induced by oxygen, light, and transition metal ions accumulated during life. Interestingly the content of melanin in the human RPE decreases with age [5] and the remaining melanin exhibits significant oxidative modifications [6]. Our previous studies showed that RPE melanosomes isolated from 60–90 years old human donors were phototoxic [7]. In another studies we showed that melanosomes isolated from bovine RPE (BMs) irradiated with visible light lost their antioxidant properties and even became prooxidant [8, 9]. RPE as metabolically active post-mitotic cells accumulate the age pigment lipofuscin. It is believed that lipofuscin contains lipid peroxidation products and their cross-links with proteins [10]. On the other hand the reported total content of proteins in purified RPE lipofuscin granules was rather low [11]. One of the key roles of RPE cells is phagocythosis of photoreceptor outer segments, which are rich in polyunsaturated fatty acids [12]. Melanosomes in the RPE, by fusion with the age pigment lipofuscin, form complex granules called melanolipofuscin [13]. It is tempting to speculate that in such a complex system, lipid hydroperoxides, originating from the lipofuscin component, could chemically modify the melanin component. It is possible that transition metal ions present in the melanin would accelerate such oxidative process. The aim of this research was to examine the effect of selected transition metal ions on melanin susceptibility to chemical modification induced by organic peroxides. Tert-butyl hydroperoxide (TBH) was used as an oxidizing agent due to the insolubility of these lipid components in water. Synthetic melanin obtained by 3,4-dihydroxyphenylalanine (DOPA) autooxidation (DMa) and BMs were analyzed.

## Materials and Methods

### Reagents

Racemic mixture of 3,4-Dihydroxy-D-β-phenylalanine and 3,4-Dihydroxy-L-β-phenylalanine (D,L-DOPA), TBH, Chelex-100, and dialysis bags were from Sigma–Aldrich Chemie Gmbh (Steinheim, Germany). EDTA (tetrasodium salt) was from British Drug Houses Ltd (Poole, UK). Potassium chloride, potassium dihydrogen phosphate, disodium hydrogen phosphate dodecahydrate, zinc sulphate heptahydrate, cupric sulphate pentahydrate, ferrous sulphate heptahydrate, methanol, and chloroform were from Standard Co. (Lublin, Poland). Sodium chloride, 35–38% hydrochloric acid, sodium hydroxide, and 98% sulfuric acid were from Polish Chemical reagents (POCH), Gliwice, Poland.

All chemicals were reagent grade or better and used as supplied. Buffer solutions, made of water deionized by a millipore system (Millipore S A. 67120 Molsheim, France), were treated with Chelex-100 to remove traces of metal ions.

### Preparation of Melanosomes and Liposomes

Cow eyes obtained from a local slaughterhouse, were transported on ice and immediately processed to isolate the pigmented non-tapetal portion of their RPE and neural and photoreceptor parts of retinas. RPE melanosomes were isolated and purified as previously described [14], except that two-step sucrose gradient (1 and 2 M), rather than multistep sucrose gradient was used during melanosome ultracentrifugation. Purified melanosomes, suspended in a small amount of phosphate-buffered saline (PBS) (pH 7.4; 0.01 M phosphates), were de-aerated by blowing a stream of argon above their surface for 1 h, tightly closed and stored on ice for further use. Retinal lipids were extracted from neural and photoreceptor parts of retinas by the Folch method [15]. Retinal lipids were quantified by determination of the dry mass of measured volume of their chloroform solution. Chloroform extract of retinal lipids was divided into portions containing about 100 mg of lipids, placed in glass bottles, dried by blowing a stream of argon and stored in −80 °C until further use. For liposome preparation the lipid portion was dissolved in argon-saturated chloroform and transferred to glass tube to make lipid film. The lipid film was dried for at least 2 h under vacuum pump. Then argon-saturated PBS was added in 5–7 small portions so that the final concentration of lipids was 50 mg/ml. After addition of all portions of PBS the tube with lipids was heated on water bath to 37 °C and shaken on Vortex shaker. The container with liposomes was blown with argon, tightly closed, stored on ice, and used no later than 3 days after preparation.

### Preparation of Synthetic Eumelanin

Synthetic model of eumelanin was prepared by DOPA autooxidation [16]. In brief, 25 g of D,L-DOPA was dissolved in 5 l of deionized water treated with Chelex-100. The solution was alkalized to pH 8.0 by addition of 25% ammonia and continuously stirred and air-bubbled for 3 days. Every several hours, the pH was adjusted to 8.0 by addition of appropriate amounts of 25% ammonia. For three more days the solution was stirred without air-bubbling, then acidified to pH 2.5 by addition of concentrated hydrochloric acid and centrifuged at 4040 g for10 min. The obtained precipitate was washed 8 times with about 4 l of acidified Chelex-100 treated deionized water (pH 2.5), and once with Chlex-100 treated non-acidified deionized water (pH ~ 5.5). The precipitate was then suspended in about 0.5 l of such water, placed in a dialysis bag (~ 12,000 u) and dialyzed for 12 days against 5 l of Chelex-100 treated deionized water changing the water four times. Melanin was quantified by determination of its dry mass.

### Electron Paramagnetic Resonance (EPR) Spectroscopy

All EPR measurements were performed using Bruker EMX-AA EPR spectrometer (Bruker BioSpin, Rheinstetten, Germany). EPR spectroscopy at 77 K was used for melanin quantification in bovine RPE melanosomes (BMs), for determination of iron and copper in melanosomes and synthetic melanin and for monitoring of progress of degradation of these melanins. Melanin in melanosomes was quantified using synthetic melanin standard prepared by DOPA autooxidation as previously described [5, 8]. Melanin degradation was monitored by EPR measurements of melanin samples at two pH values–7.4 and 0. For measurement at pH 7.4, 0.1 ml aliquot of the sample was diluted twice with PBS and frozen in liquid nitrogen. The EPR measurements at pH 0 were carried out in order to minimize effects of bound to melanin metal ions on melanin EPR signal. For this purpose 0.1 ml of sample was acidified by 10% (v/v) of concentrated hydrochloric acid, incubated for half hour, centrifuged at 20,817 g for 5 min and suspended in 1 M hydrochloric acid. The sample was washed three times using 1 M HCl by its resuspension and centrifuging as described above. Finally the sample was suspended in 0.2 ml of 1 M HCl and frozen in liquid nitrogen. For melanin measurements the apparatus settings were as follows: center field 336.1 mT, sweep width 7 mT, microwave power 33 µW, modulation amplitude 0.305 mT, time constant 327.68 ms and sweep time 41.94 s. Iron and copper were measured at pH 7.4. Iron was measured at following parameters: center field 158.49 mT, sweep width 70 mT, microwave power 5.3 mW, modulation amplitude 0.805 mT, time constant 327.68 ms and sweep time 41.94 s. For copper measurements the apparatus settings were as follows: center field 300.00 mT, sweep width 100 mT, microwave power 5.3 mW, modulation amplitude 0.805 mT, time constant 327.68 ms, and sweep time 41.94 s. Both at melanin measurements and at metal ions measurements ten spectra were averaged.

### Preparation of Metal Complexes with Melanosomes and Synthetic Melanin

The suspension of bovine RPE melanosomes (BMs) in PBS was centrifuged at 20,817 g for five min and suspended in deionized distilled Chelex-100 treated water. The washing of BMs with water was repeated twice. Finally, BMs were suspended in water at the equivalent melanin concentration 4 mg/ml (as determined by EPR spectroscopy). Measured portions of melanin suspension was further diluted to make the final concentration of melanin 2 mg /ml. The diluted suspension was acidified to pH 5 by hydrochloric acid and 0.025 M solution of zinc sulphate, cupric sulphate, or ferrous sulphate in 10^−4^ M sulfuric acid was added. The amount of added solution of metal salt was such that the final concentration was 358 µM. Taking into account atomic mass and final melanin concentration 2 mg/ml the mass percentage of metals in melanin was 1% in case of iron, 1.14% in case of copper and 1.17% in case of zinc. After introduction of selected metal salts, the pH was adjusted to 7.4 by drop-wise addition of 1 M NaOH. The samples were then stored overnight at room temperature in dark. After storage the pH was readjusted to 7.4 by measured addition of 1 M NaOH. Control BMs without added metal ions were prepared similarly as BMs with metal ions except the addition of metal salts. Ferrous sulphate was dissolved in argon-saturated 10^−4^ M sulfuric acid and used in day of preparation. Iron (II) salt was used instead iron (III) salt because ferric ions would demand acidification to pH 2, at which very little binding of iron to melanin occurs. However, we have shown by EPR spectroscopy that iron (II) is oxidized to iron (III) immediately after addition to melanin. Metal complexes with the synthetic melanin (DMa) were prepared similarly as metal complexes with BMs except PBS removal was not needed because the stock solution of DMa was in water.

### Degradation of Melanosomes and Synthetic Melanin by TBH

Bovine RPE melanosomes (BMs) and DMa with and without complexed transition metal ions were degraded at 37 °C in closed glass bottles. During degradation the samples were gently stirred. The reaction mixtures contained 1 mg/ml melanin (BMs or DMa) and 1 M TBH. To stabilize pH, the reaction mixture also contained 37% (v/v) PBS pH 7.4 diluted in water. Control samples without melanin were prepared to test the stability of TBH at 37 °C and to check whether TBH disturbed measurements of melanin optical absorption. At selected time intervals, 0.1 ml aliquots were taken for measurements of melanin, iron and copper EPR signals. These aliquots were treated as described above. At selected degradation times, greater amounts of samples were taken for further experiments. For this purpose TBH was removed from melanin samples. In the case of BMs samples three times washing of the melanosomes with distilled Chelex-100 treated water was sufficient. On the other hand DMa samples were lyophilized to remove TBH. DMa samples lyophilized immediately after addition of TBH were treated as control samples and were called in further part of this work nondegraded DMa and indicated in figures as DMa after 0 h of degradation.

### Measurements of Optical Absorption of Melanin

Optical absorption of melanin was measured in 0.05 M phosphate buffer pH 6.8 and in 1 M NaOH. 12.82 µl aliquot of sample containing 1 mg/ml melanin was added to either 0.5 ml phosphate buffer or 0.5 ml NaOH, shaken on Vortex shaker for 37 s, placed in 1 cm quartz optical cuvette and optical absorption of the sample was measured exactly after one min of sample preparation. To reduce light-scattering, the measured absorbance at 800 nm was subtracted from all values of absorbance at other wavelenghts. Optical absorption was integrated in the spectral range 350–550 nm.

### Analysis of Size of Melanin Particles

The size of nondegraded and degraded DMa particles without and with transition metal ions was determined by dynamic light scattering (DLS) using Malvern Zetasizer Nano S particle size analyzer, (Malvern, UK). DMa samples were diluted in deionized distilled filtered water to concentration 0.1 mg/ml and volume 1 ml.

### Atomic Force Microscopy (AFM) Analysis

For AFM analysis small droplets of samples were placed on a freshly cleaved mica surface and incubated for 30 min at room temperature. The samples were then gently washed with fresh PBS to remove any unattached melanin granules. Measurements were performed in PBS at room temperature using a sharp silicon nitride probe (SNL type, Bruker Probes) with a nominal tip radius of 2 nm and with experimentally determined spring constant of 0.32 N/m. Images were obtained in Tapping AC mode. Size analysis of the melanosomes was assessed by determining the long axis of the granules at longer end and short axis at the shorter end. Roughness values of melanin granules were determined using NanoScope Analysis 1.6 software (Bruker) and are presented as Rq referring to the root mean square. AFM experiments were conducted using a BioScope Catalyst AFM (Bruker, Karlsruhe, Germany).

### Oxygen Consumption Measurements

For oxygen consumption measurements the same EPR spectrometer was used as for direct EPR spectroscopy. Oxygen consumption was measured by EPR oximetry using mHCTPO as an oxygen-sensitive spin probe according to a method described elsewhere [8, 17]. Sample was irradiated with 404–510 nm light (24 mW/cm^2^) at room temperature. 0.2 ml samples typically contained 5 mg/ml liposomal lipids from bovine retinas. The apparatus settings were as follows: center field 337.67 mT, sweep width 0.3 mT, microwave power 1.06 mW, modulation amplitude 0.006 mT, time constant 40.960 ms and sweep time 5.243 s. The net oxygen consumption, accompanying lipid peroxidation, was achieved by subtraction of oxygen consumption rate in samples containing only melanin from the rate of oxygen consumption in complete samples containing both melanin and lipids.

### FOX Assay

FOX assay [18] was used to monitor TBH concentration during degradation of melanin, to determine the residual TBH in lyophilized melanin and for direct measurements of lipid peroxidation. In all of these, FOX reagent 10 x diluted in methanol with 4.4 mM butylated hydroxytoluene (BHT) was used. The diluted FOX reagent was stored on ice and used in assays the same day it was prepared. The stock solution, containing 2.5 mM ammonium ferrous sulfate and 1 mM xylenol orange in 250 mM sulfuric acid was stored in fridge and used not later than within 90 days. To monitor TBH concentration during melanin degradation aliquot of reaction mixture was diluted 5000× in PBS. To 0.1 ml of such diluted sample 0.9 ml of FOX reagent was added. After 0.5 h incubation the samples were centrifuged at 20,000 g for 10 min and the supernatant absorbace was measured at 560 nm. Residual TBH in lyophilized melanin was determined after re-dissolving of the melanin samples. TBH in these samples was determined similarly as during melanin degradation except aliquots of these samples were diluted in PBS 4× instead of 5000×. Lipid hydroperoxide accumulation was measured in samples irradiated with 402–508 nm (83 mW/cm^2^) light at 4 °C and for selected samples also at 22 °C. Sample was gently stirred during irradiation. Samples typically contained 5 mg/ml liposomal lipids from bovine retinas. At selected time intervals, 0.25 ml of sample aliquots were taken for Folch extraction [15]. Extraction mixture contained 0.4 mM BHT. 0.2 ml of chloroform extract was dried by nitrogen stream. Then 0.1 ml of chloroform and 0.9 ml of FOX reagent was added and after that samples were treated as in above described.

## Results

**Fig. 1:**
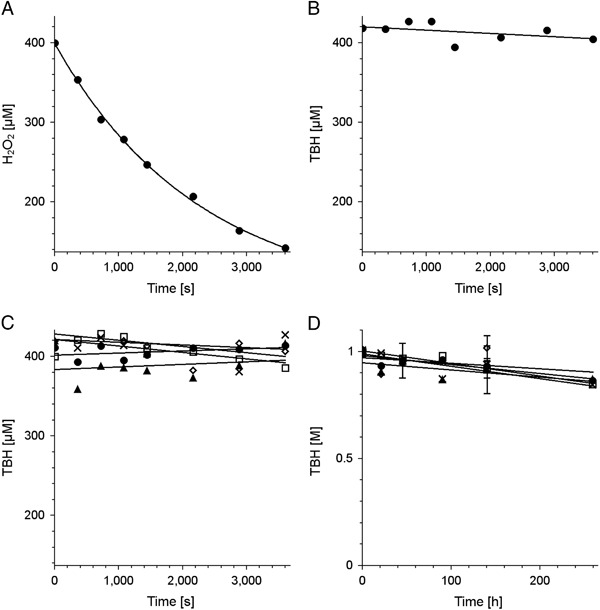
Changes in concentration of hydrogen peroxide **a** or of TBH **b**–**d** in samples containing BMs **a**, **b** or DMa **c**, **d**. *Open squares*—control sample without melanin, *filled circles*—control melanin without ions, *crosses*—melanin with zinc, *filled triangles*—melanin with copper, *open diamonds*—melanin with iron

Unlike hydrogen peroxide (Fig. 1a), TBH, at the concentation used, was not efficiently decomposed either by BMs (Fig. 1b) or by the DMa (Fig. 1c). The decrease in concentration of TBH during melanin degradation was negligible (Fig. 1d).

**Fig. 2:**
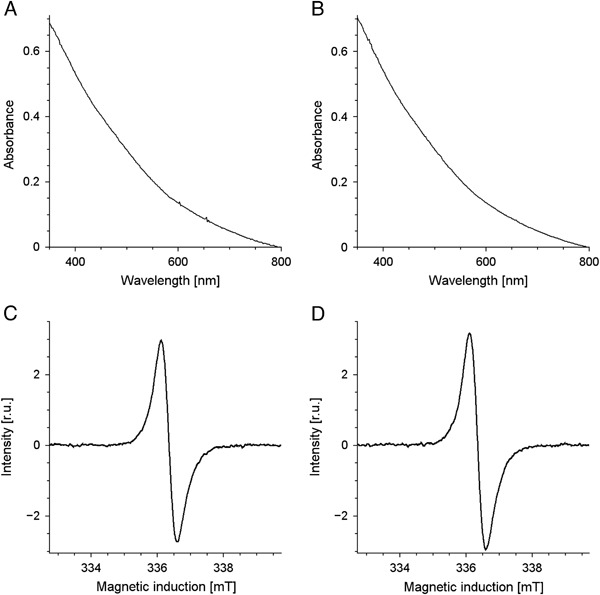
UV-vis spectra of DMa diluted to 0.025 mg/ml in 1 M NaOH (**a**, **b**) and EPR spectra of DMa in 1 M HCl, diluted to 0.5 mg/ml (**c**, **d**) untreated with TBH (**a**, **c**) or immediately after addition of TBH (**b**, **d**)

**Fig. 3:**
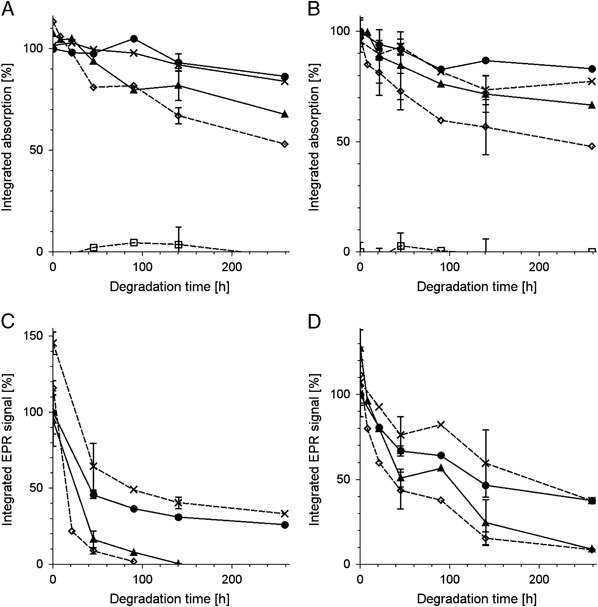
Time-dependent changes of normalized integrated absorption **a**, **b** and normalized integrated EPR signal **c**, **d** of 1 mg/ml DMa incubated with 1 M TBH at 37 °C. **a**, **b**–DMa diluted to 0.025 mg/ml in 0.05 M Na/K phosphate buffer pH 6.8 **a** or in 1 M NaOH **b**, **c**–DMa diluted to 0.5 mg/ml in PBS pH 7.4, **d**–DMa in 1 M HCl, diluted to 0.5 mg/ml. *Open squares* and *broken line*—control sample without melanin, *filled circles* and *continuous line*—control melanin without ions, *crosses* and *broken line*—melanin with zinc, *filled triangles* and *continous line*—melanin with copper, *open diamonds* and *broken line*—melanin with iron

**Fig. 4:**
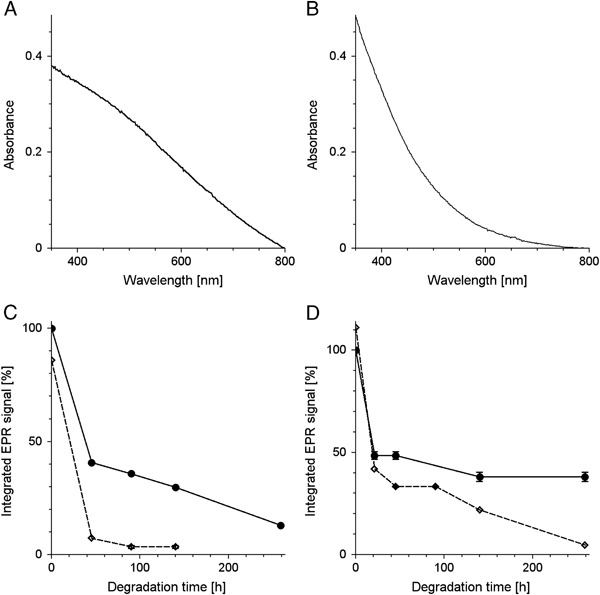
**a**, **b**–UV-vis spectra of control BMs **a** and BMs with 1% (w/w) iron **b** incubated at concentration 1 mg/ml with 1 M TBH at 37 °C for 140 h and then diluted to 0.025 mg/ml in 1 M NaOH. **c**, **d**—time-dependent changes of normalized integrated EPR signal of 1 mg/ml BMs incubated with 1 M TBH at 37 °C. **c**–BMs diluted to 0.5 mg/ml in PBS pH 7.4, **d**–BMs in 1 M HCl, diluted to 0.5 mg/ml. *Filled circles* and *continous line*—control melanosomes without exogenous ions, *open diamonds* and *broken line*—melanosomes with 1% (w/w) of exogenous iron

**Fig. 5:**
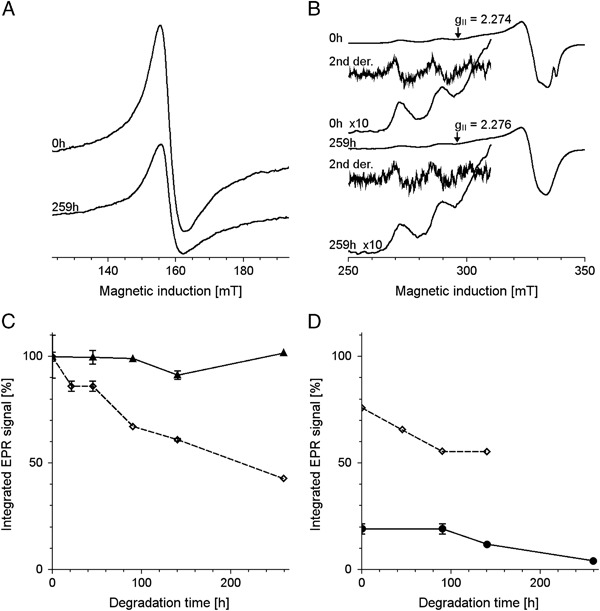
**a**, **b** – EPR spectra of iron (III) **a** and copper (II) **b** complexed with DMa diluted to 0.5 mg/ml in PBS pH 7.4 immediately and after 259 h incubation with TBH. **c**, **d**—time-dependent changes of normalized integrated EPR signal of iron (*open diamonds* and *broken line* or *filled circles* and *continuous line*) and copper (*filled triangles* and *continuous line*) complexed with DMa **c** or BMs **d**. *Filled circles* and *continuous line*—control melanin without added ions, *filled triangles* and *continuous line*—melanin with copper, *open diamonds* and *broken line*—melanin with iron

No significant changes of melanin absorption and of melanin EPR signal were observed immediately after addition of TBH to melanin (Fig. 2). On the other hand long-time incubation of DMa with 1 M TBH caused gradual decrease of optical absorption of melanin and its EPR signal (Fig. 3). Iron and copper significantly accelerated such changes while zinc had practically no effect. The effect of iron and copper on EPR signal of melanin was especially pronounced in samples containing PBS as solvent (Fig. 3c). On the other hand, the best correlation between the changes in the optical absorption and in the EPR signal of melanin was observed when UV-vis measurements were carried out in 1 M NaOH (Fig. 3b) and EPR measurements were done in 1 M HCl. Long-time incubation of BMs with 1 M TBH also caused gradual decrease of melanin EPR signal (Fig. 4c, d) and iron significantly accelerated this process (Fig. 4c). It is worth noting that the decrease of the EPR signal of melanin was faster for control BMs (Fig. 4c, d) than for control DMa (Fig. 3c, d). Optical spectrum of control BMs without exogenous ions incubated with 1 M TBH for 140 h and then diluted to 0.025 mg/ml in 1 M NaOH was mainly a result of light scattering (Fig. 4a) while such spectrum of BMs with 1% (w/w) of exogenous iron treated in the same way (Fig. 4b) resembled optical absorption spectrum of soluble melanin such as DMa (Fig. 2a). Comparison of the initial EPR signal of iron (III) in control BMs (*filled circles* and *contionous line* in Fig. 5d) with initial EPR signal of iron (III) in DMa after enrichment of melanin with 1% (w/w) iron (*open diamonds* and *broken line* in Fig. 5c), indicates that control melanosomes contain 0.2% of endogenous iron. Similar comparison of BMs with 1% (w/w) added iron (*open diamonds* and *broken line* in Fig. 5d) with DMa with 1% (w/w) iron indicates that BMs after enrichment with iron contained only 0.76% of iron bound to melanin. Incubation with 1 M TBH of DMa with 1% (w/w) iron, control BMs or BMs with 1% (w/w) added iron caused time-dependent decrease of iron (III) EPR signal (Fig. 5a, c and d). On the other hand, such incubation of DMa with copper didn’t lead to any measurable changes in the intensity of copper signal (Fig. 5b, c). Moreover, the interaction of TBH with melanin resulting in melanin degradation was not accompanied by any significant changes in the magnetic parameters of the Cu(II)-melanin signal. Thus the *g*_II_ value and the hyperfine splitting (*A*_║_) for copper complexed with nondegraded DMa are respectively 2.274 ± 0.009 and 510 ± 2 MHz, while in the case of copper complexed with degraded melanin the corresponding values are 2.276 ± 0.008 and 504 ± 2 MHz. The only difference between EPR signal of copper (II) bound to nondegraded melanin and melanin degraded with TBH for 259 h was that the perpendicular component of copper EPR signal (B ≈ 326.8 mT i.e., *g*_┴_ ≈ 2.066) was partly split in the case of nondegraded melanin (Fig. 5b).

The solutions of DMa with iron and copper incubated with TBH for 259 h and then diluted to 0.1 mg/ml in water were much lighter than those of control DMa and DMa with zinc treated the same way. The effect was especially pronounced in the case of DMa with iron.

**Fig. 6:**
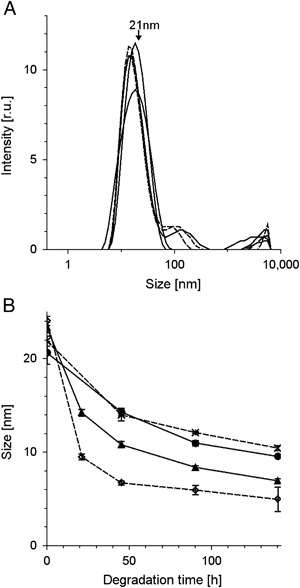
Effect of TBH on average size of DMa particles: **a**–size distribution of undegraded 0.1 mg/ml control DMa in water without ions measured in five repetitions. **b**–time-dependent changes of particle size of DMa incubated at concentration 1 mg/ml with 1 M TBH. Size measurement was performed after DMa dilution to 0.1 mg/ml in water. *Filled circles* and *continuous line*—control melanin without ions, *crosses* and *broken line*—melanin with zinc, *filled triangles* and *continous line*—melanin with copper, *open diamonds* and *broken line*—melanin with iron

DLS analysis of DMa revealed that its average particle size was 21 nm (Fig. 6a). Although initial size of DMa particles with ions seemed to be a bit greater than the size of DMa without ions, iron and copper significantly accelerated TBH-induced reduction in the particle size of DMa (Fig. 6b). The effect was particularly sizeable for iron-containing melanin samples; thus after 140 h of incubation with TBH of DMa with iron, the particle size of melanin decreased to 5 nm while the particle size of control DMa decreased to 10 nm.

**Fig. 7:**
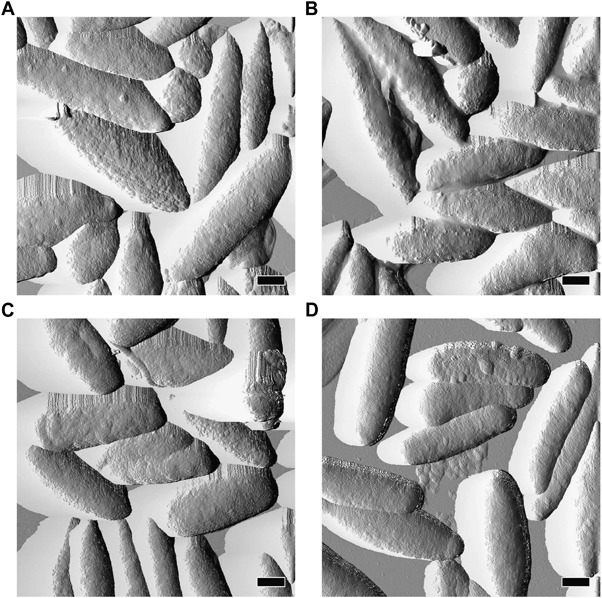
Representative AFM amplitude images of control melanosomes **a**, melanosomes degraded with TBH for 90 h **b**, non-degraded melanosomes with iron **c** and melanosomes with iron degraded with TBH for 90 h **d**. *Scale bars* represent 500 nm

AFM analysis revealed that chemical degradation of melanosomes induced changes in the shape and size of the pigment granules. Typical shape of eumelanosomes is considered as ‘ellipsoidal’ with two different axes [19]. Such shape was observed for control melanosomes, melanosomes subjected to chemical degradation and melanosomes with addition of iron ions but without degradation, whereas for melanosomes degraded in the presence of iron, the shape became more cigar-like (Fig. 7). Such morphological changes of the melanosomes subjected to TBH-induced oxidation, catalyzed by iron ions, could be due to a weaker binding interaction of melanin nanoaggregates with the protein matrix in the area of short melanosome axis [20]. Average sizes of the studied melanosomes are shown in Table 1. In our previous work we have shown that in vitro photoaging of bovine RPE melanosomes induces morphological changes of the pigment granules, exposing melanin nanoaggregates [21]. In this study, we observed similar effects induced by chemical degradation. The effect was quantitatively characterized by measuring the roughness of melanosomes. Since melanin nanoaggregates are small and very homogeneous compared to lipid fragments [22], the roughness values of melanosomes with the exposed nanoaggregates, should be lower. Indeed roughness values of degraded melanosomes were noticeably smaller than those of control melanosomes (Table 1).

**Table 1:** Effect of TBH-induced degradation on morphological parameters of melanosomes

Sample^a^	EPR melanin signal (%)^b^	Long axis (nm)	Short axis (nm)	Roughness (nm)
BMS_0h	100	2456 ± 128	737 ± 41	4.81 ± 0.07
BMS_90h	43	2304 ± 98*	705 ± 35*	3.95 ± 0.03*
BMS_Fe_0h	111	2543 ± 158	715 ± 33	4.63 ± 0.06
BMS_Fe_90h	33	2078 ± 60**	579 ± 49**	3.38 ± 0.06**

^a^ Sample names refer to experimental conditions: BMS_0h—control melanosomes; BMS_90h—melanosomes degraded with TBH for 90h; BMS_Fe_0h—non-degraded melanosomes with iron; BMS_Fe_90h—melanosomes with iron degraded with TBH for 90h

^b^ Intensity of EPR signal of melanin free radicals

*Statistically significant vs. BMS_0h sample; **statistically significant vs. BMS_Fe_0h sample. For all values *P* < 0.001

**Fig. 8:**
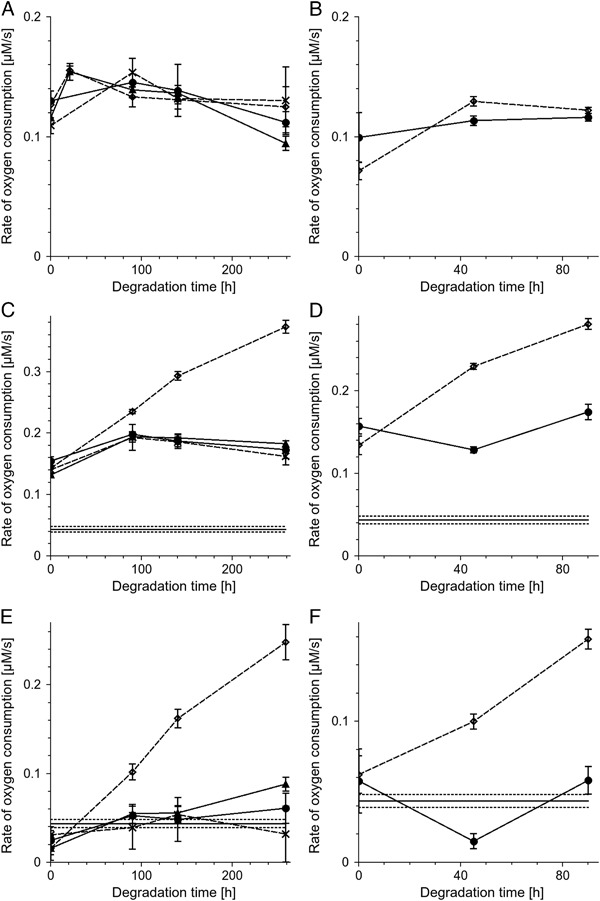
Initial rate of oxygen consumption induced by irradiation with blue light (404–510 nm, 24 mW/cm^2^) as a function of time of melanin incubation with TBH. Samples contained 0.5 mg/ml melanin without lipids **a**, **b** or 0.5 mg/ml melanin with 5 mg/ml liposomal lipids extracted from bovine retinas **c**, **d**. **e, f**–net oxygen consumption accompanying lipid peroxidation. The melanin is DMa **a**, **c**, **e** or BMs **b**, **d**, **f**. *Filled circles* and *continuous line*—control melanin without ions, *crosses* and *broken line*—melanin with zinc, *filled triangles* and *continous line*—melanin with copper, *open diamonds* and *broken line*—melanin with iron. *Horizontal continuous line* indicates the rate of oxygen consumption in samples containing only 5 mg/ml retinal lipids and *horizontal dotted lines* indicate standard deviation of oxygen consumption in this system

Oxygen consumption induced by irradiation of 0.5 mg/ml DMa or BMs without lipids with light (404–510 nm, 24 mW/cm^2^) was slow and did not depend on metal ion content or degradation state of melanin (Fig. 8a, b). The consumption rate was in the range 0.09–0.16 µM/s for DMa (Fig. 8a) or 0.06–0.13 µM/s for of BMs (Fig. 8b). Liposomes, containing 5 mg/ml lipids from bovine retinas irradiated with light under the same experimental conditions as described above, consumed oxygen with the rate 0.044 ± 0.005 µM/s (Fig. 8c–f). Without iron, oxygen photoconsumption in complete systems containing liposomal lipids and melanin was only slightly higher than oxygen photoconsumption in control samples containing melanin without lipids (Fig. 8c, d). Addition of iron ions had no effect on oxygen photoconsumption if nondegraded melanin was examined. On the other hand, iron ions significantly accelerated oxygen photoconsumption in samples containing lipids and melanin modified by its interaction with TBH. The corresponding rates of oxygen photoconsumption were: 0.37 ± 0.01 µM/s for DMa incubated with TBH for 259 h (Fig. 8c) and 0.281 ± 0.007 µM/s for BMs incubated with TBH for 90 h (Fig. 8d). The net oxygen photoconsumption rate accompanying lipid peroxidation was slightly lower in samples containing nondegraded DMa regardless metal ion content (Fig. 8e) and did not exceed 0.04 µM/s. Such melanin-related inhibition was not observed in samples of nondegraded BMs (Fig. 8f). Neither melanin without ions significantly affected oxygen photoconsumption (Fig. 8e, f). The effect of copper became appearent only after 259 h of the incubation time of DMa with TBH; in such samples, oxygen photoconsumption increased to 0.088 ± 0.008 µM/s (Fig. 8e). Both iron containing DMa and iron containing BMs incubated with TBH strongly accelerated net oxygen photoconsumption and this effect increased with degradation state of melanin (Fig. 8e, f). The net oxygen photoconsumption was 0.25 ± 0.02 µM/s in sample with iron containing DMa incubated with TBH for 259 h and 0.158 ± 0.007 µM/s in sample with iron containing BMs incubated with TBH for 90 h.

**Fig. 9:**
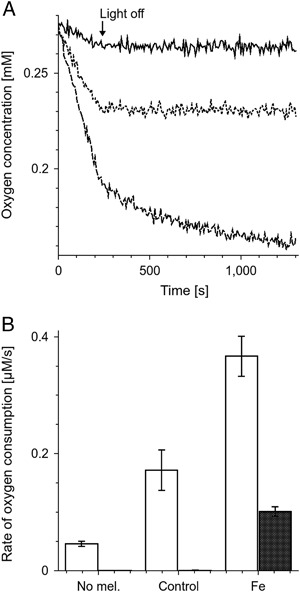
**a**–time-dependent changes of oxygen concentration in samples irradiated with blue light for 240 s and then incubated in dark. Samples contained only 5 mg/ml liposomal lipids (*continuous line*), 5 mg/ml liposomal lipids and 0.5 mg/ml DMa without ions degraded for 259 h (*dotted line*) or 5 mg/ml liposomal lipids and 0.5 mg/ml iron containing DMa degraded for 259 h (*broken line*). *Arrow* indicates the time when the irradiation was stopped. **b**–Initial rate of oxygen consumption induced in samples containing only 5 mg/ml liposomal lipids (No mel.), 5 mg/ml liposomal lipids and 0.5 mg/ml DMa without metal ions degraded for 259 h (Control) or 5 mg/ml liposomal lipids and 0.5 mg/ml iron containing DMa degraded for 259 h (Fe). *Empty bars*—samples irradiated with blue light, *filled bars*—samples incubated in dark after such irradiation. Irradiation conditions as in Fig. 8

In samples containing only lipids and in samples containing melanin modified by TBH for 259 h but without iron, consumption of oxygen stopped immediately after irradiation of the samples was discontinued (Fig. 9). On the other hand, in samples containing lipids and degraded melanin with iron, consumption of oxygen continued in the dark with the rate 0.102 ± 0.008 µM/s.

FOX assay indicated that lyophilized DMa samples re-dissolved in water to final concentation 2 mg/ml contained about 1 mM residual TBH.

**Fig. 10:**
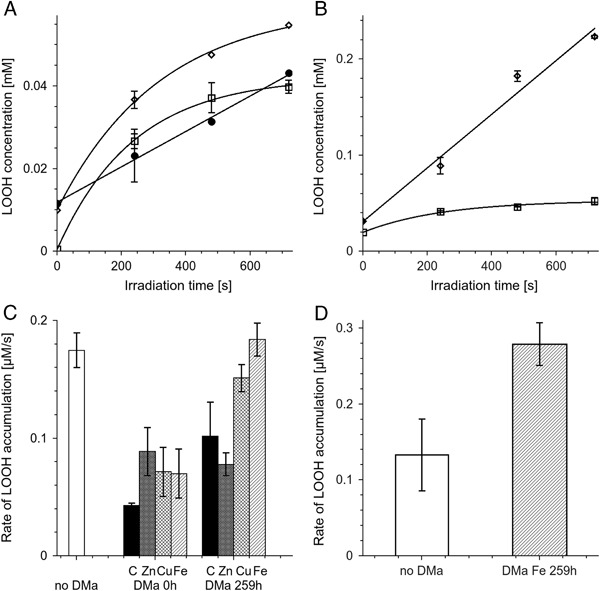
**a**, **b**—time-dependent changes of lipid hydroperoxide concentration in samples containing 5 mg/ml liposomal lipids without melanin (*open squares*), with 0.5 mg/ml of nondegraded control DMa without ions (*filled circles*), or with 0.5 mg/ml of iron containing DMa degraded for 259 h (*open diamonds*). **c**, **d**—initial rate of lipid hydroperoxide accumulation in control containing only 5 mg/ml liposomal lipids (no DMa), lipids with 0.5 mg/ml undegraded DMa (DMa 0 h) or lipids with 0.5 mg/ml DMa degraded with TBH for 259 h (DMa 259 h) without C or with Zn, Cu, or Fe ions. Samples were irradiated with 402–508 nm (83 mW/cm^2^) light at 4 °C **a**, **c** or at 22 °C (**b**, **d**)

Direct measurements of lipid hydroperoxide accumulation at 4 °C (LOOH) by FOX assay showed that the initial rate of light-induced LOOH accumulation in system containing only 5 mg/ml liposomes was 0.17 ± 0.015 µM/s (Fig. 10a, c). Nondegraded control DMa without metal ions inhibited LOOH accumulation so that the observable rate was 0.043 ± 0.002 µM/s. The effect of nondegraded DMa with metal ions was comparable. The rate of LOOH accumulation in samples with such melanin was in the range 0.05–0.11 µM/s (Fig. 10c). Samples with control DMa without metal ions, degraded by TBH for 259 h, exhibited light-induced LOOH accumulation with the rate 0.10 ± 0.029 µM/s. The effect of degraded DMa with zinc was comparable with the effect of undegraded DMa with metal ions. Both copper containing and iron containing DMa lost their inhibitory capacity after 259 h incubation with TBH. LOOH accumulation rate in samples containing DMa with copper, degraded for 259 h was 0.15 ± 0.011 µM/s. The rate of LOOH accumulation in samples containing DMa with iron, degraded for 259 h was 0.18 ± 0.014 µM/s.

At 22 °C, the initial rate of light-induced LOOH accumulation in samples containing only 5 mg/ml liposomes was 0.13 ± 0.05 µM/s (Fig. 10b, d). On the other hand, iron-containing DMa degraded for 259 h increased LOOH accumulation to 0.28 ± 0.03 µM/s

## Discussion

Although the actual mechanism of the interaction of hydrogen peroxide with the melanosome constituents has not been clarified, the ability of melanosomes to decompose hydrogen peroxide was previously demonstrated [9]. As a result, accumulation of hydrogen peroxide in melanosomes or in pigment granules containing melanin component is not likely. On the other hand, neither BMs nor DMa catalyze decomposition of TBH. The observed small decrease of TBH concentration during melanin degradation was probably due to stoichiometric interaction of TBH with melanin rather than to a catalytic process. To achieve reasonably fast degradation of melanin, TBH concentration was in high excess compared to melanin concentration. Extrapolating the results obtained in this study to processes that may occur in the aging human RPE, where complex pigment granules such melanolipofuscin are formed [13], it can be assumed that organic hydroperoxides, derived from polyunsaturated lipids, accumulate in lipofuscin and melanolipofuscin granules. Under such conditions, melanin in melanolipofuscin granules could be exposed to substantial concentration of lipid hydroperoxides. It can be postulated that under such conditions oxidative modifications of the melanin component may occur. We have previously shown that oxidative modifications of melanosomal melanin can also be induced by prolong aerobic irradiation of the pigment granules with intense UVA or visible light [6]. However, there are distinct changes between photooxidation of melanin and its chemical modification induced by organic hydroperoxides. Thus while the initial stages of melanin photooxidation were accompanied by a transient increase in integrated optical absorbance of melanin and its EPR signal intensity [8, 9], no such changes were observed during incubation of melanin with TBH (Figs 2 and 3). It appears that the interaction of melanin with TBH mostly induces irreversible changes in the melanin structure and properties. On the other hand the transient increase in the optical absorption and content of free radicals, induced in melanin during the early irradiation times, can be interpreted as reversible photooxidation of melanin subunits. Signifficant acceleration of TBH-induced degradation of melanin by redox active metal ions, such as copper or iron, suggests involvement of alkoxy radical (LO^•^) in melanin degradation. This radical can be formed in Fenton type reaction:

$${\rm{LOOH}} + {\rm{Mel{-}Fe}}\left( {{\rm{II}}} \right) \to {\rm{Mel{-}Fe}}\left( {{\rm{III}}} \right) + {\rm{O}}{{\rm{H}}^ - } + {\rm{L}}{{\rm{O}}^ \bullet }$$

or

$${\rm{LOOH}} + {\rm{Mel{-}Cu}}\left( {\rm{I}} \right) \to {\rm{Mel{-}Cu}}\left( {{\rm{II}}} \right) + {\rm{O}}{{\rm{H}}^ - } + {\rm{L}}{{\rm{O}}^ \bullet }$$

where LOOH is TBH (or lipid hydroperoxide) and Mel-Fe(II), Mel-Fe(III), Mel-Cu(I) and Mel-Cu(II) are respectively iron (II), iron (III), copper (I) copper (II) complexes with melanin. Similar Fenton type reaction has previously been demonstrated for copper complexes with synthetic melanin [23]. Iron (II) and copper (I) can be formed by melanin-mediated reduction of iron (III) and copper (II). Although the involvement of peroxyl radical (LOO^•^) cannot be ruled out:

$${\rm{LOOH}} + {\rm{Mel{-}Fe}}\left( {{\rm{III}}} \right) \to {\rm{Mel{-}Fe}}\left( {{\rm{II}}} \right) + {{\rm{H}}^ + } + {\rm{LO}}{{\rm{O}}^ \bullet }$$

$${\rm{LOOH}} + {\rm{Mel{-}Cu}}\left( {{\rm{II}}} \right) \to {\rm{Mel{-}Cu}}\left( {\rm{I}} \right) + {{\rm{H}}^ + } + {\rm{LO}}{{\rm{O}}^ \bullet }$$

the interaction of lipid hydroperoxides with Fe(III) and Cu(II) is much slower than such interaction with the reduced form of the metal ions. Acceleration by iron and copper of DMa bleaching indicates that redox active metal ions accelerated TBH-induced melanin degradation. Incubation of DMa with TBH also increases melanin dispersion (Fig. 6b). Acceleration by iron and copper of TBH-induced disaggregation of melanin manifested as its increased dispersion, confirms the correlation between melanin degradation and its dispersion. Unlike control BMs (Fig. 4a), BMs with exogenous iron (Fig. 4b) became solubilized due to 140 h degradation with TBH. It indicates that iron ions accelerated also melanosome solubilization. Such solubilization can result from substantial damage of melanin polymer and/or of structural melanosomal proteins.

EPR measurements of melanin in 1 M HCl after three times washing with this acid are more reliable than such measurements in PBS without washing. This is because washing with 1 M HCl facilitates removal of metal ions complexed with melanin and, as a result, the EPR signal of such melanin is not affected by paramagnetic or diamagnetic metal ions. Moreover, at low pH the comproportionation equilibrium between the fully reduced and oxidized melanin subunits and melanin radicals is significantly shifted towards the diamagnetic constituents, which minimizes the inducible melanin EPR signal [24], which enables a more accurate monitoring of melanin degradation. On the other hand, EPR measurements in PBS at pH 7.4 enabled qualitative analysis of the release of metal ions during degradation. Diamagnetic metal ions, such as zinc, increase melanin EPR signal because of stabilization of melanin radical by forming chelate complexes with these ions [24]. Thus weaker effect of zinc ions on EPR signal of degraded melanin (Fig. 3c), may suggest a reduction in the melanin binding sites, resulting from its altered redox capacity. On the other hand, paramagnetic metal ions, such as copper and iron quench melanin EPR signal due to dipole-dipole interaction of unpaired spins of these ions with melanin radicals [25]. Faster decrease of melanin EPR signal with copper and iron in PBS than in HCl suggests that release of copper and iron was slower than the reduction in the content of melanin radicals. It can be concluded that during the TBH-induced degradation of melanin, the percentage of paramagnetic metal ions in relation to melanin radicals, increased. Optical absorption measurements of melanin in 1 M NaOH are more reliable than such measurements in 0.05 M Na/K phosphate buffer pH 6.8 because in 1 M NaOH melanin functional groups are deprotonated and at least synthetic melanin is fully solubilized. The situation is different at pH 6.8; under these conditions some of the melanin functional groups are still protonated and the state of melanin dissociation might be affected by metal ions. On the other hand at pH 6.8 melanin is chemically much more stable than in alkaline media, where it may autooxidize quite fast.

TBH-induced degradation of control BMs (Fig. 4) was faster than such degradation of DMa (Fig. 3) because synthetic melanin was almost free of iron, while BMs contained 0.2% (w/w) of endogonous iron accumulated in melanin during cow lifetime. Lower efficiency of iron binding by BMs (Fig. 5d) than by DMa (Fig. 5c) may result from the granular nature of melanosomes, which limits the accessibility of melanin binding sites to metal ions. In addition, it cannot be ruled out that the content of metal ion binding sites in synthetic melanin is higher than that in melanosomal melanin. Time-dependent decrease of iron EPR signal in synthetic melanin or bovine melanosomes (Fig. 5a, c and d) suggests gradual release of iron ions during melanin degradation. On the other hand, lack of significant changes of the EPR signal of copper (II) bound to melanin during TBH-induced degradation of DMa (Fig. 5b, c) indicates that copper was not released during melanin degradation. It may suggest that binding of copper ions by melanin involves different ligands than binding of iron ions. Negligible changes in parallel components of copper EPR signal suggest that predominant mode of copper binding by melanin does not change during melanin degradation [26].

Higher initial level of lipid hydroperoxides in sample with DMa resulted from presence of residual TBH in melanin samples. Residual TBH content in lyophilized DMa might slightly influence the observed results, however all examined DMa samples contained similar content of residual TBH so comparison of the effect of metal ions or of degradation was reliable.

Transition metal ions used in this research and TBH-induced degradation of DMa and BMs did not influence significantly their photoreactivity (Fig. 8a, b). Light-induced photoconsumption of oxygen observed in complete systems with lipids and melanins (Fig. 8c, d) was a superposition of oxygen consumption due to lipid peroxidation and melanin oxidation. The net effect (Fig. 8e, f), was estimated by subtraction of oxygen consumption in samples containing melanins without lipids from oxygen consumption in complete samples with melanins and lipids. Comparison of the net oxygen consumption in samples with nondegraded DMa with oxygen consumption in samples containing lipids without melanosomes led to a conclusion that both nondegraded DMa without metal ions and nondegraded DMa with transition metal ions exhibited similar antioxidant action (Fig. 8e). The conclusion was confirmed by direct measurements of lipid hydroperoxides (Fig. 10a, c). It is apparent that nondegraded control DMa without metal ions, under the conditions used behaved as the most efficient antioxidant. Lack of measurable antioxidant action of bovine melanosomes (Fig. 8f) could be explained by their granular nature which limited the effective radius of the melanin interaction with reactive oxygen species. Antioxidant action of native BMs was previously observed in similar system [9] but in the cited experiments the BMs concentration was substantially higher. The net oxygen consumption in the system with control or zinc containing DMa degraded by TBH was similar to that with undegraded DMa (Fig. 8e). However, direct measurements of lipid hydroperoxide accumulation indicate that DMa degraded by TBH might slightly lose its antioxidant capacity (Fig. 10c). Both iron containing melanins degraded by TBH exhibited prooxidant action which increased with oxidation state. Prooxidant effect of DMa with Cu was also observed for DMa degraded for 259 h but this effect was lower than in case of DMa with iron. There are two possible mechanisms of lipid peroxidation in sample with liposomes and iron containing DMa degraded for 259 h. The first is photoperoxidation sensitized by products and metabolites of the visual pigment rhodopsin such as retinoids that are probably present in the chloroform extract from bovine retinas. The second mechanism may be chemical peroxidation induced by Fenton type reaction [10]. To separate these mechanisms, oxygen consumption was measured during irradiation of the samples and right after the irradiation was discontinued (Fig. 9). In samples containing only liposomal lipids or liposomal lipids and degraded DMa without ions, termination of the irradiation resulted in an immediate slowing down the oxygen consumption to zero. On the other hand samples with liposomal lipids and degraded DMa with iron, behaved differently, i.e., oxygen consumption continued, albeit at reduced pace, even after termination of the sample irradiation. This dark oxygen consumption, after termination of the sample irradiation, can be explained assuming that lipid peroxidation continues in the dark, being initiated by Fenton type reaction. On the other hand, if lipid hydroperoxide (LOOH) accumulation was measured at 4 °C then chemical peroxidation was strongly inhibited. Thus there was no significant difference in LOOH accumulation between sample without melanin and the sample with iron containing DMa degraded for 259 h (Fig. 10c). However, at 22 °C the degraded DMa with iron accelerated LOOH accumulation more than by factor of two. The data indicate that, while light-induced lipid peroxidation in sample containing only retinal lipids, is mainly due to photosensitized peroxidation, in sample with lipids and degraded DMa with iron, the chemical peroxidation might be a dominant process. Lipid hydroperoxide accumulation was measured in samples irradiated by more intense light than during oxygen consumption measurements and thus lipid peroxidation measured directly in control system containing only lipids was higher than lipid peroxidation determined by oxygen consumption in such sample. Therefore lipid peroxidation at 22 °C in samples with degraded DMa with iron should also be faster than that determined by oxygen consumption. It is important to stress that the observed LOOH accumulation is a superposition of lipid peroxidation and decomposition of LOOH. In the presence of iron and at 22 °C LOOH decomposition could be fast, which explains the lower rate of LOOH accumulation than net oxygen consumption in irradiated system with degraded DMa with iron. The prooxidant action of degraded DMa and BMs with iron suggests that iron in such system is redox active. Similar prooxidant action in the presence of iron was previously observed in case of human melanosomes degraded by light [27]. It is tempting to speculate that similar conditions might develop in situ in the aging human RPE. When iron containing melanosomes undergo fusion with lipofuscin granules, then melanin in the formed melanolipofuscin can be exposed to substantial local concentration of lipid hydroperoxides present in the age pigment lipofuscin. As a result of a long-time interaction of LOOH with melanin, degradative modifications of the melanin could occur. Since the degraded melanin with iron is an efficient prooxidant agent as shown in this study, such processes could increase the risk of oxidative stress in the aging RPE and this, in turn, could contribute to the development of age-related macular degeneration. It could be of particular relevance to this study that RPE and choroidal melanosomes from donors with of age-related macular degeneration exhibit elevated iron content in comparison to melanosomes from healthy donors [28].

## Conclusions

This study has demonstrated that the organic hydroperoxide TBH can induce oxidative degradation of synthetic eumelanin and melanosomes from bovine RPE. Melanosomes degraded with TBH exhibited distinct morphological changes with reduced surface roughness. The degradative process is significantly accelerated by redox active metal ions, particularly iron. The synthetic melanin, subjected to TBH-induced degradation, losses its antioxidant capacity. Iron-enriched synthetic melanin and melanosomes become prooxidant after degradation with TBH. It can be postulated that the accumulating in the aging human RPE melanolipofuscin provides conditions that facilitate oxidative modification of the melanin component, which may increase the risk of oxidative stress in the outer retina.
